# Supplementary material for: Modeling glioblastoma heterogeneity as a dynamic network of cell states
Source: Mol Syst Biol. 2021 Sep 16;17(9):e10105. doi: 10.15252/msb.202010105 (PMC8444284; doi:10.15252/msb.202010105)
Supplement: Supplementary file 6 — Source Data for Figure 5 [file MSB-17-e10105-s004.zip › Figure5A_sourcedata/GSEA_3071/hallmarks_stateIII.GseaPreranked.1623742540963/HALLMARK_ANGIOGENESIS.html]

Details for gene set HALLMARK\_ANGIOGENESIS[GSEA]

|  || Dataset | state33071 |
| Phenotype | NoPhenotypeAvailable |
| Upregulated in class | na\_neg |
| GeneSet | HALLMARK\_ANGIOGENESIS |
| Enrichment Score (ES) | -0.6454521 |
| Normalized Enrichment Score (NES) | -2.5414238 |
| Nominal p-value | 0.0 |
| FDR q-value | 0.0 |
| FWER p-Value | 0.0 |
Table: GSEA Results Summary

  

Fig 1: Enrichment plot: HALLMARK\_ANGIOGENESIS      
 Profile of the Running ES Score & Positions of GeneSet Members on the Rank Ordered List

  

| PROBE | GENE SYMBOL | GENE\_TITLE | RANK IN GENE LIST | RANK METRIC SCORE | RUNNING ES | CORE ENRICHMENT || 1 | VAV2 |  |  | 963 | -0.277 | -0.2231 | No |
| 2 | FSTL1 |  |  | 1254 | -0.293 | -0.2728 | No |
| 3 | MSX1 |  |  | 1715 | -0.320 | -0.3637 | No |
| 4 | FGFR1 |  |  | 1770 | -0.323 | -0.3505 | No |
| 5 | TIMP1 |  |  | 2038 | -0.337 | -0.3906 | No |
| 6 | JAG2 |  |  | 2082 | -0.340 | -0.3731 | No |
| 7 | ITGAV |  |  | 2942 | -0.414 | -0.5582 | No |
| 8 | CCND2 |  |  | 3037 | -0.425 | -0.5468 | No |
| 9 | JAG1 |  |  | 3424 | -0.486 | -0.6048 | Yes |
| 10 | NRP1 |  |  | 3562 | -0.532 | -0.5954 | Yes |
| 11 | COL5A2 |  |  | 3726 | -0.636 | -0.5839 | Yes |
| 12 | VCAN |  |  | 3741 | -0.660 | -0.5324 | Yes |
| 13 | TNFRSF21 |  |  | 3763 | -0.691 | -0.4800 | Yes |
| 14 | STC1 |  |  | 3770 | -0.702 | -0.4228 | Yes |
| 15 | APP |  |  | 3779 | -0.710 | -0.3655 | Yes |
| 16 | LUM |  |  | 3861 | -0.870 | -0.3135 | Yes |
| 17 | POSTN |  |  | 3885 | -1.025 | -0.2338 | Yes |
| 18 | COL3A1 |  |  | 3918 | -1.418 | -0.1235 | Yes |
| 19 | SPP1 |  |  | 3920 | -1.508 | 0.0023 | Yes |
Table: GSEA details [plain text format]

  

Fig 2: HALLMARK\_ANGIOGENESIS: Random ES distribution      
 Gene set null distribution of ES for **HALLMARK\_ANGIOGENESIS**
